# Use of Robotic Devices for Gait Training in Patients Diagnosed with Multiple Sclerosis: Current State of the Art

**DOI:** 10.3390/s22072580

**Published:** 2022-03-28

**Authors:** Sagrario Pérez-de la Cruz

**Affiliations:** Department of Nursing, Physical Therapy and Medicine, University of Almería, Carretera de Sacramento s/n, La Cañada de San Urbano, 04120 Almería, Spain; spd205@ual.es; Tel.: +34-950-21-45-74

**Keywords:** multiple sclerosis, robotic therapy, rehabilitation, gait, movement

## Abstract

Multiple sclerosis (MS) is a neurodegenerative disease that produces alterations in balance and gait in most patients. Robot-assisted gait training devices have been proposed as a complementary approach to conventional rehabilitation treatment as a means of improving these alterations. The aim of this study was to investigate the available scientific evidence on the benefits of the use of robotics in the physiotherapy treatment in people with MS. A systematic review of randomized controlled trials was performed. Studies from the last five years on walking in adults with MS were included. The PEDro scale was used to assess the methodological quality of the included studies, and the Jadad scale was used to assess the level of evidence and the degree of recommendation. Seventeen studies met the eligibility criteria. For the improvement of gait speed, robotic devices do not appear to be superior, compared to the rest of the interventions evaluated. The methodological quality of the studies was moderate–low. For this reason, robot-assisted gait training is considered just as effective as conventional rehabilitation training for improving gait in people with MS.

## 1. Introduction

Multiple sclerosis (MS) is a disease affecting the central nervous system (CNS) characterized by inflammation, demyelination, and axonal damage of the brain and spinal cord [1]. It mainly affects the young adult population, usually between 20 and 40 years of age, and especially women, by a three to one ratio compared to men [2]. At present, according to the Spanish Federation for the Fight against multiple sclerosis and the Spanish Society of Neurology, there are 47,000 people suffering from this disease in Spain, 700,000 in Europe, and 2,500,000 worldwide [3]. It is one of the most frequent causes of neurological disability in young adults [4]. Its course cannot be predicted and can vary greatly from person to person [3]; however, 50% of those affected are unable to walk independently 15 years after onset [4]. Although altered sensation (45%), motor impairment (40%), and visual disturbances (20%) are usually the first symptoms of this disease, MS is highly heterogeneous, with a diversity of symptoms that limit the patient’s quality of life. Thus, the symptoms, their severity, and the order of onset can vary across patients; therefore, the social and occupational repercussions may differ from one person to another [5].

Reduced mobility, and specifically difficulty walking, is a frequent and early disorder which has a significantly negative impact on these patients [6]. Gait impairment results from the combination of several common symptoms present in MS, such as fatigue, weakness, sensory disturbances, spasticity, ataxia, and loss of balance [7,8]. Several studies have stated that up to 90% of patients with MS experience some degree of reduced mobility [6,9,10], with difficulties in walking from the first decade since the onset of the disease, requiring some assistance to walk [6,7]. Although gait impairment has been observed to increase as disease and disability progress, patients with Expanded Disability Status Scale (EDSS) < 3.5 may also have significant gait impairment [8,9,10,11]. 

The gait pattern of patients with MS is characterized by lower speed, shorter stride length, and longer time in double stance phase, as well as lower amplitude in the ankle dorsiflexion movement during the stance phase and greater plantar flexion during the moment of initial heel contact, in comparison with gait patterns in the normal population [12,13,14,15]. In addition, they have less muscle activity (tibialis anterior and gastrocnemius) in signs of significant pyramidal involvement [12,13,14,15]. All these symptoms imply significant economic and social burdens, since they lead to a progressive loss of work productivity and personal autonomy [6,7].

Among the different therapeutic options available to clinicians in the process of motor rehabilitation in MS patients, several robotic devices exist (treadmill with or without suspension of body weight, training with electromechanical assistance, exoskeletons—Lokomat, for example, among others), aimed at maintaining optimal conditions or preventing motor deterioration in these patients. Robotics is defined as the application of devices with electronic or computerized systems designed to perform human functions [11,13]. A therapeutic robot is a system that detects the user’s movements, using this information to adjust parameters and provide visual and sensory feedback to the patient [16]. These are noninvasive devices that are easy to control, with low risk for the patient and with good treatment effectiveness [16]. Robotic rehabilitation offers certain advantages for rehabilitation: reproducibility, task-oriented programs, quantified progression, playful environment, reduced energy costs, and greater functional independence for both the patient and the therapist [17,18]. Therefore, the use of this therapeutic option continues to undergo significant development and growth within the field of physical rehabilitation [18,19].

The aim of this systematic review was to identify studies that have established a relationship between gait rehabilitation in MS patients using robotic devices and to evaluate the efficacy of their use in the treatment of gait for these patients.

## 2. Materials and Methods

A systematic review was conducted of the existing literature regarding the use of robotics in the treatment of people diagnosed with multiple sclerosis for intervention in the gait rehabilitation process. The information was compiled by searching the following databases: PubMed, Scopus, Web of Science, Dialnet, PEDro, and Lilacs. The following search terms were used: multiple sclerosis, robotics, robotic therapy, therapy, movement, gait, and randomized controlled trials.

The PICO strategy was followed as a principle for the selection criteria of this study: (P) persons diagnosed with MS with gait impairment; (I) robotic systems for ambulation; (C) use of robotic devices versus another treatment approach or none; (O) improvement of gait parameters, distances traveled, energy expenditure, as well as psychological benefits.

### 2.1. Selection Process

First, a general search with the terms: “multiple sclerosis” [Mesh] and (“robotics” or “robotic therapy” or “therapy”) and (“rehabilitation” or “therapy” or “movement”) and “gait” was performed in each database consulted. Inclusion criteria were as follows:

- Participants with a diagnosis of multiple sclerosis, in any of its clinical variants, as well as any degree of disability or severity of deficit, time since diagnosis, age, and sex.
- Participants over 18 years of age.
- The intervention employed robotic interventions for the purpose of gait training.
- Randomized clinical trials (intervention), clinical cases, or any work involving human intervention.
- Studies conducted in the last five years (2017–2021) and showing the results obtained.
- Studies in English, Portuguese, or Spanish.

Abstracts, editorials, and notes were discarded, as were systematic reviews. Once the results of a general search were obtained, a selection was made based on the title and abstracts to verify that they still met the inclusion criteria. Duplicates were eliminated. Finally, the full text of each article was examined, and those that met the criteria determined in the selection process were selected.

### 2.2. Data Extracted

For each study, a summary was made with the following information: sample size (both experimental and control group, if any), study aim, technology used in the intervention, methodology (sessions/week, duration of sessions, etc.), evaluation (measurement scales), and results (improvements or changes in the evaluated population). These data were extracted by the author using the Consort 2010 statement for randomized controlled clinical trials whenever possible [20].

### 2.3. Level of Evidence

To evaluate the level of evidence and methodological quality of the selected studies, the Jadad scale [21] was used, which considers randomization, blinding of the patients and the investigator to the intervention (double-blind study), and the description of any losses that may have occurred during the trial. A Likert scale was used (from 0 to 5 points, with 5 being the maximum score), assigning quality to the randomized clinical trial. Quality is considered “poor” if the score obtained is lower than 3 points. 

The PEDro scale [22] consists of 11 items, which consider scientific rigor, assessing the following aspects: selection criteria, randomization of subjects, allocation concealment, baseline compatibility, blinding of subjects, blinding of therapists, blinding of evaluators, adequate follow-up, intention-to-treat analysis, between-group analysis, mean scores, and variability. All the above except the item assessing the selection criteria was used to calculate the final score of an article (maximum: 10 points).

## 3. Results

**Figure 1:**
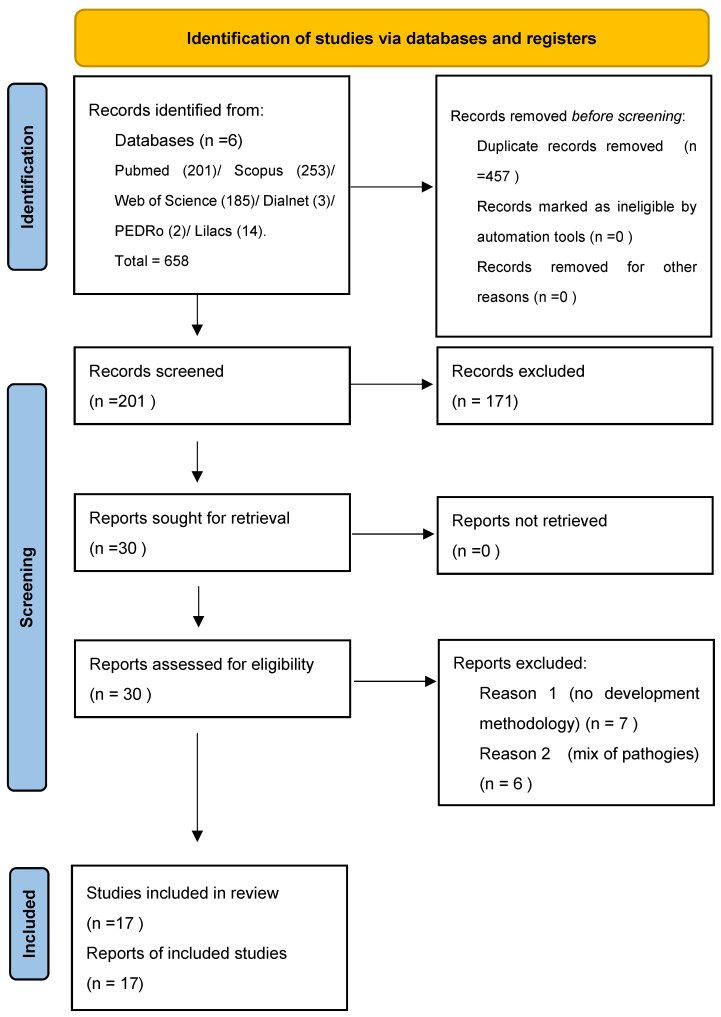
PRISMA flowchart.

After searching the different databases, 658 records were obtained. After eliminating duplicate articles, 231 records were obtained, of which 201 were discarded according to the criteria for considering studies for this review. In addition, references were reviewed from the reference list of retrieved articles for possible additional, relevant references; however, no new records were obtained. Ultimately, 17 articles that met all the inclusion criteria were selected and added to this review. The flowchart of the systematic review is shown in Figure 1.

### 3.1. Studies Included

Seventeen RCTs [23,24,25,26,27,28,29,30,31,32,33,34,35,36,37,38,39] (537 participants) were included. All articles were published in English. Details of each RCT selected for this review are presented in Table 1. Information regarding the populations, interventions, durations, and outcomes of the various studies are also shown in Table 1.

### 3.2. Participants

The ages of the participants in the included studies ranged from 18 to 70 years, with most of the samples being considered young adults. In terms of sex, the studies showed a small difference between the number of men and women, with the number of female subjects being slightly higher than the number of males, except for the studies by Nierdermeier [26] and Puyuelo-Quintana [31], with only one patient. In relation to the number of participants per sample in each of the studies analyzed, the number of participants was not very high, the largest being that of Staudi [33] with a total of 98 participants compared to 14 participants in the studies by Niedermeier [26] and Puyuelo-Quintana [31], which were divided between brain damage and multiple sclerosis. The mean of all the studies was 31.58 participants. Although some samples have a large number of participants, they insufficient for extrapolating the results to the total number of people with MS.

Most of the studies selected participants with MS aged between 21 and 65 years. MS is characterized by being a common disease in middle-aged adults when beginning work. Therefore, studies should be carried out on the ages where this pathology can influence their quality of life, since the loss of functionality in middle-aged adults can hinder their development in the spheres of personal, social, and working life, thus indirectly affecting society at large.

### 3.3. Type of Intervention

Nine of the studies [23,24,25,27,28,29,30,33,34] included two comparison groups: the experimental group, the group where the intervention to be evaluated was performed, and the control group, the group with which the results were compared, and a conservative treatment was applied. The remaining studies were pre–post studies or comparisons between two types of interventions (comparative studies) [26,31,34,35,36,37,38,39].

### 3.4. Outcome Measures 

Different scales have been used in studies to assess motor, cognitive, and functional aspects. 

At the motor level, the following measures were used: the timed get-up-and-go (TGUG) [23,24,30,32,34,35,36], Tinetti [23,25,31], 25-foot walking test [25,29,33,34,37], six-minute walking test [25,27,29,30,32,34,35], Modified Motricity Index for Lower Limbs [25], gait parameters such as cadence and speed [25,29,32,37,39], the two-minutes walking test [28,39], Functional Ambulatory Category [26], Barthel [28], Rivermead Mobility Index [28], and the 10-step stair test [27,31].

The following scales were used for cognitive assessment: the Hamilton Rating Scale for Depression (HRSD) [23,24,27], the Coping Orientation to Problem Experience (COPE) [24], the MSIS-29 [29], mental quickness, and brain connectivity [27,30,38].

The functional domain was evaluated using the Expanded Disability Severity Scale (EDSS) [23,27], Functional Independence Measure (FIM) [23,24,25,27], Berg Balance Scale [24,29,32,39], Modified Ashworth Scale [24,25,33], VAS [28], Quality-Of-Life Index [25,32], and fatigue severity scale [28,33,34,36,37]. In one of the studies, blood protein concentration was evaluated [27].

### 3.5. Results Obtained

All the studies analyzed have shown benefits in the use and employment of robotic-assisted therapy in patients with multiple sclerosis. Specifically, the study by Russo [27] indicated that the experimental group (training with Lokomat) improved all the values evaluated, whereas the control group (conventional training) only showed improvement in the TGUG test. Conversely, Calabró et al. [24] indicated no changes in their study in relation to the values obtained in the Berg Balance Scale and the TGUG test. Another aspect to be valued in some studies is the fact that outcomes are evaluated during the intervention, upon completion of the intervention, and at follow-up; the time period for the latter was variable, ranging from 30 days after the end of the intervention [23,27] to three months later [29,33]. In the studies that continued to evaluate patients after the end of the intervention, the results obtained (improvements in motor gait values) were maintained over time.

### 3.6. Methodological Quality, According to the PEDro Scale

The scores obtained on the PEDro scale are shown in Table 2. Ten [25,26,27,28,29,30,33,34,35,39] of the seventeen studies obtained a score of 8 out of 11, whereas the studies by Puyuelo-Quintana [31], Łyp [32], and Druzbicki [37] fall below medium quality, with a score of 3 points. 

Overall, 82.35% of the selected studies showed a high level of quality, obtaining a score equal to or higher than 6, whereas 17.65% obtained a score equal to or lower than 5 and were considered to have a low level of quality. The criteria that had the worst score were criteria 3 (the assignment was concealed), 5 (all subjects were blinded), and 6 (all therapists who administered the therapy were blinded).

### 3.7. Quality of Studies According to Jadad

Table 3 shows the qualitative summary of the selected studies. Six of the studies analyzed showed acceptable quality (score equal to or higher than 3) [25,33,34,36,38,39], one of the studies was double-blinded (blinding of both the evaluator and the participants) [26], and when used, the blinding method was not described. Four papers [30,31,37,38] were intervention studies; however, the patients were not randomized, and therefore all those who met the inclusion/exclusion criteria were included in the study. In general terms, the methodological quality of these studies is considered poor, implying a bias that may affect the results obtained.

## 4. Discussion

The use of robotics has undergone important development and continuous advances, growing in its field of action and in its scientific basis, since new technologies applied to gait can increase and favor motor learning or provide professionals with objective measures for its treatment. The present review revealed that an acceptable number of studies exist related to the use of robotic devices in the gait rehabilitation of patients diagnosed with multiple sclerosis. Both gait-assisting robots and specific robotic devices that assist and control deficits that these patients present in their ambulation pattern have been studied. Although there is a steady growth of studies in this field, many are considered mere prototypes for this type of subject and pathology.

It is important to consider that robotic training involves complex systems that interact with the musculoskeletal and nervous systems. However, the intrinsic dynamics of the lower limbs are known to influence movement; therefore, an understanding of robotic training systems is important. In relation to gait control, basic rhythmic movements that originate in neurons located at the spinal cord level (central pattern generators—CPGs) can elicit activity, which stimulates motor learning of the musculoskeletal system. This action presents in motor learning an improvement in muscle tone, strength, balance, trunk control, and functional capacity, all achieved by continuous repetitions [29,40]. Factors that positively influence patient outcome are intensity, precocity, repeatability, and specificity in task-oriented training [40,41]. With their constant and symmetric lower extremity trajectories, robotic devices provide many of the proprioceptive inputs that can increase cortical activation and stimulation of the CGP to improve motor function. The use of robotic-assisted gait training (RAGT) enables repetition of specific and stereotyped movements to acquire a correct and reproducible gait pattern under conditions of balance and symmetry, early initiation of treatment using body weight-bearing activity, and patient safety with reduced fear of falling, minimizing the intervention of a therapist [7,9,29,42]. It is also important to highlight the energy control that retraining their gait with these devices entails for a patient with multiple sclerosis, since energy expenditure during activity will be reduced after training with the robotic device [13,34].

Some skepticism may currently exist regarding technological devices that can be applied in the clinical setting [42]. Previous studies [43,44] have suggested that these beliefs originated from a lack of evidence, incomplete description (reporting the number of degrees of joint freedom as well as the anatomical structures involved are important to explain the complexity of the movements performed), and limitations of studies on robotic-assisted treatment, and there may be deficiencies in the replicability of such studies in clinical practice, or fear that they may replace the work of professionals [42,43]. However, the aim of introducing this aid is to improve treatment options [43]. Several factors influence this fact: Engineers develop technological innovation systems, whereas clinicians need time and training to be able to learn how to use and develop the new device in daily clinical practice [42].

Reviewing the existing literature regarding the use of robotic devices in neurological pathology, most studies investigated stroke rehabilitation, with special attention to patients in a chronic phase of the disease [42,45,46,47,48,49,50,51,52,53]. However, the literature has also reported that early mobilization and interventions in this regard reduce the duration of hospitalization and improve the degree of disability [46,47]. Despite this evidence, it is reasonable to think that robotic devices, by performing passive or assisted movements, may be relevant in the acute and subacute phases of the disease, although less so in the chronic phase. Only one study [27] shows the effects in the chronic phase, together with the prescription of antispasmodic medication, achieving encouraging results, considering the possibility of improving their efficacy by using a joint intervention including robotics and medication. However, the results vary in degenerative pathologies, such as MS. The fact that MS is a degenerative disease with an unpredictable and irregular course, in many cases characterized by the presence of outbreaks of myelin destruction, which causes an increase in the patient’s involvement, may cause the benefits of treatment to vary, preventing the results from being statistically significant for robotic therapy. The studies analyzed [23,24,25,26,27,28,29,30,31,32,33,34,35,36,37,38,39] have shown that robotic devices are useful in rehabilitation; however, it would be advisable to verify that this continues to be the case in the different phases of the disease. An example of this line of work is the studies in which patients have received post-treatment follow-up [48,49]. Although it has been shown that the intensive training performed by robotic devices boosts neuronal plasticity [52], the number of repetitions proposed in the studies (no more than 45–50 min in each session) is insufficient to stimulate neuroplasticity. There is considerable unanimity regarding the type of pathology and its development (relapsing-remitting MS) in the application of therapeutic options. However, this would be an interesting point to study and show results in applications for other types of pathologies and degrees of severity. Thus, it would be possible to define the ideal characteristics for the application of robotic therapy in multiple sclerosis in order to obtain greater benefits.

Another common symptom affecting 8 to 55% of people with MS is depression [52]. This clinical condition shows improvement after the practice of physical activity, both in individuals with and without MS [54,55,56,57]. In addition to this symptom, favorable results are also found in fatigue, anxiety, and quality of life for those whose who regularly practice exercise [58]. In the studies analyzed [22,23,24,25,26], changes at the psychological level are observed, but the results vary depending on the scale used in the measurement, and therefore it would be convenient to analyze the sensitivity of the scales used to determine which measures can detect small changes in the psychological parameters of patients with MS.

Although incorporating these aids into the therapy of MS patients can also have certain disadvantages, such as the high cost of their acquisition and maintenance, and the poor personal bond that is created between therapist and patient [24,26,27,28,33], it is important to consider that the use of these devices involves a series of indispensable requirements. Thus, patients should have adequate cognitive skills to understand verbal commands, and cooperation between both parties is required in order to obtain the best possible performance and effectiveness.

One of the limitations found in this study was the scarcity of existing studies on the subject during the last 10 years, other than pilot studies, although an interesting line of work is being developed. In addition, the heterogeneity of the methodologies applied, the sample sizes used, and the lack of homogeneity in the intervention protocol may have influenced the results and benefits attributable solely to robotic therapy, making it difficult to extrapolate the results to the MS population as a whole. It is important to highlight that the results of the Puyuelo-Quintana study [31] could not be extrapolated due to the small sample, although it is interesting to take into account his report due to the fact that he proposed the use of a robotic gait system in acquired and degenerative neurological pathologies.

The long-term effects of the application of robotic gait therapy in people with MS, whether the effects of robotics on ambulation vary according to the type of MS, and the degree of disability of the participants have not been evaluated, and therefore it is not known how long the changes in the sample may last. For future studies, long-term follow-up studies should be developed. Similarly, the effects of dosage (time in the sessions, duration of the proposed programs, etc.) on the changes observed in the studies should be investigated to optimize resources and carry out relatively homogeneous research that will enable the development of more complete studies. The studies analyzed present a moderate methodological quality, and therefore an improvement in the scientific methodology is recommended to avoid possible biases in the results.

## 5. Conclusions

The use of robotics has undergone significant development and continues to do so, growing in its field of action and in its scientific basis for the development of therapeutic strategies applied to patients with multiple sclerosis. These devices allow both professionals and patients to be motivated and involved in the rehabilitation process, due to the benefits revealed so far in the clinical trials analyzed. The exercises included while training with the different devices have allowed patients to obtain significant improvements in the maintenance of their motor skills. It is advisable to continue working on the design of new motor program options for these robotic systems, bearing in mind the suggestions of clinical specialists and patients with MS, so that they can be fully integrated into the rehabilitation process of these individuals. We hope that the results of this study will provide guidance to the clinician in relation to decision-making, protocol development, and the updating of guidelines.

## Figures and Tables

**Table 1:** Summary of selected contributions.

Author/Year	Study	N, EG/CG	Intervention	Outcome Measures	Results
Russo et al. [23]	Single-blind randomized trial	N = 45 EG: 30 CG: 15	EG: 6 weeks Lokomat (3 times/week, 60 min) + 12 weeks traditional training (3 times/week, 60 min) CG: 18 weeks traditional training. Measurements: beginning (T1), final (six weeks later; T2), one month later (T3)	Expanded Disability Severity Scale (EDSS); Functional Independence Measure (FIM); Hamilton Rating Scale for Depression (HRSD); TUG, Tinetti	EG improved on all scales, while CG only on TUG. GC improved at all values of T1 and T2, while EG only improved TUG at those times. At T2 and T3 there were no major differences between the two groups.
Calabró RS et al. [24]	Single-blind randomized clinical trial	N = 40 EG: 20 CG: 20	EG: Lokomat-Nanos (RAGT − VR) CG: Lokomat-Pro (RAGT + VR) 5 times/week, 8 weeks. Measurements: beginning to end of treatment	TUG, Berg, Coping Orientation to Problem Experience (COPE), FIM, Modified Ashworth scale, Hamilton Rating Scale for Depression (HRSD)	There are no differences between values obtained in Berg and TUG. In the rest of the scales, they show significant improvements (*p* < 0.05) in the use of RAGT and VR. The combined use of Lokomat treatment with virtual reality exercises improves symptoms in MS patients.
Sconza C et al. [25]	Randomized controlled crossover trial	N = 17 EG: 8 CG: 9	EG: Lokomat + physiotherapy CG: physiotherapy 5 times/week 5 weeks	25-foot walking test (T25FW), 6-minute walking test (6MWT), Tinetti; Ashworth, Modified Motricity Index for Lower Limbs, FIM, Quality-Of-Life Index, gait parameters	Both groups showed improved results, but EG improved especially in the 25FW and 6MWT trials.
Niedermeier M et al. [26]	Crossover study	N = 14 EG: 7 CG: 7	EG: Lokomat CG: Bobath principles, comprised mobilization, strengthening, and sensomotoric stimulating techniques. One Lokomat session and one conventional physiotherapy session, administered randomly to each group. Measurement at the beginning and end of each session	Personal perception questionnaire, Short version of the German Mood Survey Scale (MSS) Functional Ambulation Category (FAC)	RAGT showed significantly increased euphoria and calm after the treatment session. Affective responses between physical therapy and RAGT differed significantly in favor of RAGT in affective states.
Russo M et al. [27]	Rater-blinded, active controlled, parallel-group pilot study	N = 40 EG: 20 CG: 20	EG: Sativex + Lokomat CG: other antispasmodic + Lokomat 45 min, 3 times/week. Duration: 20 sessions. Measurement: beginning–final–30 days later	EDSS, Functional Independence Measure (FIM), MAS, NRS, 10MWT, 6-minute walking test (6MWT), Hamilton Rating Scale for Depression (HRSD), and MSQOL54. Cortical plasticity was evaluated by means of TMS methodology. Blood pressure and mean heart rate were assessed	Patients treated with Sativex and Lokomat improved gait and balance motor values, compared to patients treated with another type of antispasmodic.
Pompa A et al. [28]	Pilot, single-blind randomized controlled trial	N = 43 EG: 21 CG: 22	EG: robot-assisted gait training (RAGT), CG: conventional walking training (CWT) In the morning, 3 times/week, 4 weeks, 40 min/session. Measurement: pre- and post-intervention	2-min walking test (2MWT) Functional Ambulatory Category (FAC), Rivermead Mobility Index (RMI), Modified Barthel Index (mBI), fatigue severity scale (FSS), visual analogue scale (VAS)	Experimental group presented better results on the scales than the control group, which means that assisted gait training leads to improvement in gait.
Ziliotto N et al. [29]	Parallel-assignment, single-blinded, randomized controlled trial	N = 61 EG: 33 CG: 28	12 sessions; duration: two hours each for six weeks. EG: RAGT on a Lokomat treadmill with a duration of about 40 min CG: assisted walking on the ground. Sessions of approximately 40 min, inserted between 10 min warm-up and cool-down periods	Gait speed, assessed by the T25FWT, the 6-min walking test (6MWT), the Berg Balance Scale (BBS), and the MS impact scale-29 (MSIS-29)	The protein concentration and blood concentration values after motor treatment varied from one group to another, and an increase in protein concentration was found in EG, leading to an improvement in motor skills.
Androwis GJ et al. [30]	Pilot single-blind, randomized controlled trial	N = 10 EG: 6 CG: 4	Compared the effects of 4 weeks of REAER with 4 weeks of conventional gait training (CGT). Duration: 4 weeks, 2 times/week. Measurement: beginning–final	Functional mobility (timed up-and-go- TUG-), walking endurance (six-minute walking test- 6MWT-), cognitive processing speed (CPS; Symbol Digit Modalities Test- SDMT-), and brain connectivity (thalamocortical resting-state functional connectivity (RSFC)	REAER improved the items evaluated, due to the adaptive and integrative plasticity of the central nervous system.
Puyuelo-Quintana G et al. [31]	Cross-sectional study	N = 5 (four stroke patients and one with MS)	5 sessions of 50 min. Pre- and post-measurement, combining measurements without a device, with a device, and with different gait modes that the MAK exoskeleton allows	10-m walking test (10MWT), the Gait Assessment and Intervention Tool (G.A.I.T.) and Tinetti Performance Oriented Mobility Assessment (gait subscale) Modified QUEST 2.0 Questionnaire	The MAK exoskeleton appears to offer positive preliminary results in terms of safety, feasibility, acceptability, and use by patients.
Łyp M et al. [32]	Pilot study	N = 20 (10 males and 10 females)	A six-week-long training period with the use of robot-assisted treadmill training of increasing intensity of the Lokomat type	Difference in motion dependent torque of lower extremity joint muscles after training compared with baseline before training	The robot-assisted body-weight-supported treadmill training may be a potential adjunct measure in the rehabilitation paradigm of “gait reeducation” in peripheral neuropathies.
Straudi S et al. [33]	Parallel-group, randomized controlled trial	N = 98	EG: RAGT intervention on a robotic-driven gait orthosis (Lokomat) CG: individual conventional physiotherapy focusing on over-ground walking training performed with the habitual walking device Measurements: beginning (6 sessions) to final (12 sessions) to three months later. 3 sessions/week, two hour duration	T25FW; QoL; 6-min walking test (6MWT); Berg Balance Scale; timed up-and-go test; fatigue severity scale; Modified Ashworth Scale; Patient Health Questionnaire; Short Form Health Survey; Multiple Sclerosis Impact Scale; Multiple Sclerosis Walking Scale	The RAGT training is expected to improve mobility compared to the active control intervention in progressive MS. Unique to this study is the analysis of various potential markers of plasticity in relation with clinical outcomes, identifying the effectiveness of intensive rehabilitative interventions through the changes of clinical and circulating biomarkers of MS plasticity.
Straudi S et al. [34]	Randomized controlled trial	N = 72 EG: 36 CG: 36	EG: robot-assisted gait training (RAGT) CG: conventional therapy (CT) 12 sessions, for 4 weeks. Measurements: beginning (6 sessions) to final (12 sessions) to three months later	T25FW test, the 6-min walking test (6MWT), the Berg Balance Scale (BBS), the timed up-and-go (TUG) test, the fatigue severity scale (FSS), the Patient Health Questionnaire (PHQ), the Short Form Health Survey 36 (SF-36), the MS impact scale-29 (MSIS-29), and the MS walking scale-12 (MSWS-12)	RAGT was not superior to CT in improving gait speed in patients with progressive MS and severe gait disabilities where a positive, even transitory, effect of rehabilitation was observed.
McGibbon CA et al. [35]	An open-label, randomized, crossover trial	N = 29	Unassisted (rehab effect) performance was observed after using the device at home for 2 weeks, compared to 2 weeks at home without the device, and participants improved their ability to use the device over the trial period (training effect)	6-minute walking test (6MWT); TUG test; timed stair test (TST)	Keeogo appears to deliver an exercise-mediated benefit to individuals with MS that improved their unassisted gait endurance and stair climbing ability.
Berrozabalgoitia R et al. [36]	Randomized controlled trial	N = 36	CG: rehabilitation program consisting of weekly 1-hour individualized sessions. EG: also participated in this program in addition to a twice-weekly individualized and progressive OR gait training intervention for 3 months, aiming to reach a maximum of 40 min by the end of the 3-month period	10-meter walking test (10MWT); the Short Physical Performance Battery, the timed up-and-go (TUG) test, and the Modified Fatigue Impact Scale	The evaluated intervention could preserve gait speed and significantly improve functional mobility without increasing perceived fatigue in participants. Thus, OR exoskeletons could be considered a tool to deliver intensive practice of good-quality gait training in individuals with MS and moderate to severe gait impairments.
Drużbicki M et al. [37]	Single-group longitudinal preliminary study	N = 14	15 exoskeleton-assisted gait training sessions, reflected by the muscle strength of the lower limbs and by walking speed. Assessments were performed 4 times, that is, prior to the start of the program (T0), at the end of the physiotherapy without an exoskeleton (T1), at the end of the exoskeleton-assisted training (T2), and at 6-week follow-up (T3)	Dynamometric knee extensor and flexor strength (Biodex Pro4), postural balance, and center of pressure displacements (Zebris FMD-S), walking speed measured with the timed 25-foot walking test and fatigue (fatigue severity scale)	Individuals with MS and severe gait impairment participating in exoskeleton-assisted gait training achieved significant improvement in lower-limb muscle strength and increase in walking speed, yet the effect was not long-lasting.
Maggio MG et al. [38]	Randomized controlled trial	N = 60 EG: 30 CG: 30	The effect of semi-immersive virtual reality training (sVRT) on neuropsychological and motor recovery individuals suffering (EG) was evaluated. CG: conventional cognitive training. Measurement: beginning–final	Cognitive and motor outcomes were investigated through clinical and neuropsychological scales	A significant improvement in cognitive parameters and motor scores was observed only for EG.
Munari D et al. [39]	Randomized controlled trial	N = 17	EG: robot-assisted gait training with virtual reality CG: robot-assisted gait training without virtual reality Measurements: beginning–final (one month later)	Paced Auditory Serial Addition Test, Phonemic Fluency Test, Novel Task, Digit Symbol, Multiple Sclerosis Quality of Life-54, 2-min walking test, 10-meter walking test, Berg Balance Scale, gait analysis, and stabilometric assessment	Both forms of training led to positive influence on executive functions. However, larger positive effects on gait ability were noted after robot-assisted gait training engendered by virtual reality with multiple sclerosis.

PT: physical therapy; RAGT: robotic-assisted gait training; EG: experimental group; CG: control group; EDSS: Expanded Disability Severity Scale; FIM: Functional Independence Measure; HRSD: Hamilton Rating Scale for Depression; TUG: timed up-and-go; VR: virtual reality; RAGT: robot-assisted gait training; COPE: Coping Orientation to Problem Experience; MS: multiple sclerosis; T25FW: 25-foot walk test; 6MWT: 6-Minute Walking Test; MSS: Mood Survey Scale; FAC: Functional Ambulation Category; MSQOL54: Multiple Sclerosis Quality of Life-54; CWT: conventional walking training; 2MWT: 2-minute Walking Test; mBI: Modified Barthel Index; FSS: fatigue severity scale; VAS: visual analogue scale; BBS: Berg Balance Scale; MSIS-29: Multiple Sclerosis Impact Scale-29; CGT: Conventional Gait Training; CPS: cognitive processing speed; SDMT: Symbol Digit Modalities Test; RSFC: Resting State Functional Connectivity; 10MWT: 10-m walking test; GAIT: Gait Assessment and Intervention Tool; MSWS-12: Multiple Sclerosis Walking Scale-12; SVRT: semi-immersive virtual reality training.

**Table 2:** PEDro scale: methodological quality.

Author	1—Eligibility Criteria Were Specified	2—Subjects Were Randomly Allocated to Groups	3—Allocation Was Concealed	4—The Groups Were Similar at Baseline	5—There Was Blinding of All Subjects	6—There Was Blinding of All Therapists	7—All Assessors Blinded Who Measured at Least One Key Outcome	8—At Least One Key Outcome Was Obtained from More than 85% of the Subjects Initially Allocated to Groups	9—All Subjects Were Analyzed by “Intention to Treat”	10—The Results Are Reported for at Least One Key Outcome	11—The Study Provides Both Point Measures and Measures of Variability	Total
Russo M et al. [23]	X	X		X				X	X	X	X	7/11
Calabró RS et al. [24]	X	X		X				X	X	X	X	7/11
Sconza C et al. [25]	X	X		X			X	X	X	X	X	8/11
Niedermeier M et al. [26]	X	X		X			X	X	X	X	X	8/11
Russo M et al. [27]	X	X		X			X	X	X	X	X	8/11
Pompa A et al. [28]	X	X		X			X	X	X	X	X	8/11
Ziliotto N et al. [29]	X	X		X			X	X	X	X	X	8/11
Androwis GJ. et al. [30]	X	X		X			X	X	X	X	X	8/11
Puyuelo-Quintana G [31]	X								X		X	3/11
Łyp M et al. [32]	X							X	X		X	4/11
Straudi S et al. [33]	X	X		X			X	X	X	X	X	8/11
Straudi S et al. [34]	X	X		X			X	X	X	X	X	8/11
McGibbon CS et al. [35]	X	X		X			X	X	X	X	X	8/11
Berrozabalgoitia R [36]	X	X		X				X	X	X	X	7/11
Druzbicki M et al. [37]	X								X		X	3/11
Maggio MG et al. [38]	X	X		X				X	X	X	X	7/11
Munari D et al. (2020) [39]	X	X		X			X	X	X	X	X	8/11

Note: the sign X means that this item complies.

**Table 3:** Jadad scale: level of evidence and methodological quality of the selected studies.

Article	Was the Study Randomized?	Was the Study Described as Randomized and Blinded?	Was the Method of Double Blinding Appropriate?	Was the Method of Double Blinded Described and Appropriate?	Was There a Description of Withdrawals and Dropouts?	Total
Russo M et al. [23]	+	+	−	−	−	2
Calabró RS et al. [24]	+	+	−	−	−	2
Sconza C et al. [25]	+	+	−	−	+	3
Niedermeier M et al. [26]	+	−	+	−	−	2
Russo M et al. [27]	+	+	−	−	−	2
Pompa A et al. [28]	+	−	−	−	−	1
Ziliotto N et al. [29]	+	−	−	−	−	1
Androwis GJ. et al. [30]	+	−	−	−	+	2
Puyuelo-Quintana G et al. [31]	−	−	−	−	−	0
Łyp M et al. [32]	−	−	−	−	+	1
Straudi S et al. [33]	+	+	−	−	+	3
Straudi S et al. [34]	+	+	−	−	+	3
McGibbon CS et al. [35]	+	+	−	−	−	2
Berrozabalgoitia R et al. [36]	+	+	−	−	+	3
Druzbicki [37]	−	−	−	−	+	1
Maggio GM [38]	+	+	−	−	+	3
Munari D et al. [39]	+	+	−	−	+	3

Note: + means that it complies with that article; − means that it does not comply with that article.

## Data Availability

Not applicable.

## References

[1] Lassmann H. (2003). Axonal injury in multiple sclerosis. J. Neurol. Neurosurg. Psychiatry.

[2] de Souza-Teixeira F., Costilla S., Ayan C., Garcia-Lopez D., Gonzalez-Gallego J., De Paz J.A. (2009). Effects of Resistance Training in Multiple Sclerosis. Int. J. Sports Med..

[3] Federación Española Para la Lucha Contra la Esclerosis Múltiple (2017). FELEM. http://www.esclerosismultiple.com/.

[4] Klineova S., Lublin F.D. (2018). Clinical Course of Multiple Sclerosis. Cold Spring Harb. Perspect. Med..

[5] Oh J., Vidal-Jordana A., Montalban X. (2018). Multiple sclerosis: Clinical aspects. Curr. Opin. Neurol..

[6] Edwards T., Pilutti L.A. (2017). The effect of exercise training in adults with multiple sclerosis with severe mobility disability: A systematic review and future research directions. Mult. Scler. Relat. Disord..

[7] LaRocca N.G. (2011). Impact of walking impairment in multiple sclerosis: Perspectives of patients and care partners. Patient.

[8] Zwibel H.L. (2009). Contribution of impaired mobility and general symptoms to the burden of multiple sclerosis. Adv. Ther..

[9] Sandroff B.M., Motl R.W., Scudder M.R., DeLuca J. (2016). Systematic, Evidence-Based Review of Exercise, Physical Activity, and Physical Fitness Effects on Cognition in Persons with Multiple Sclerosis. Neuropsychol. Rev..

[10] Hemmett L., Holmes J., Barnes M., Russell N. (2004). What drives quality of life in multiple sclerosis?. QJM.

[11] Feinstein A., Freeman J., Lo A.C. (2015). Treatment of progressive multiple sclerosis: What works, what does not, and what is needed. Lancet Neurol..

[12] Cameron M.H., Nilsagard Y. (2018). Balance, gait, and falls in multiple sclerosis. Handb. Clin. Neurol..

[13] Moreno-Verdu M., Ferreira-Sanchez M.R., Cano-de-la-Cuerda R., Jimenez-Antona C. (2019). Eficacia de la realidad virtual sobre el equilibrio y la marcha en esclerosis multiple. Revision sistematica de ensayos controlados aleatorizados [Efficacy of virtual reality on balance and gait in multiple sclerosis. Systematic review of randomized controlled trials]. Rev. Neurol..

[14] Cofre-Lizama L.E., Khan F., Lee P.V., Galea M.P. (2016). The use of laboratory gait analysis for understanding gait deterioration in people with multiple sclerosis. Mult. Scler..

[15] Cameron M.H., Wagner J.M. (2011). Gait abnormalities in multiple sclerosis: Pathogenesis, evaluation, and advances in treatment. Curr. Neurol. Neurosci. Rep..

[16] Glegg S.M., Tatla S.K., Holsti L. (2014). The GestureTek virtual reality system in rehabilitation: A scoping review. Disabil. Rehabil. Assist. Technol..

[17] Rodriguez Clauido I. (2012). Entrenamiento robótico como medio de rehabilitación para la marcha. Evid. Médica Investig. Salud.

[18] Bergmann J., Krewer C., Bauer P., Koenig A., Riener R., Müller F. (2018). Virtual reality to augment robot-assisted gait training in non-ambulatory patients with a subacute stroke: A pilot randomized controlled trial. Eur. J. Phys. Rehabil. Med..

[19] Kramer A., Dettmers C., Gruber M. (2014). Exergaming with additional postural demands improves balance and gait in patients with multiple sclerosis as much as conventional balance training and leads to high adherence to home-based balance training. Arch. Phys. Med. Rehabil..

[20] Schulz K.F., Altman D.G., Moher D., CONSORT Group (2010). CONSORT 2010 Statement: Updated guidelines for reporting parallel group randomised trials. BMJ.

[21] Olivo S.A., Macedo L.G., Godotti I.C., Fuentes J., Stanton T., Magee D.J. (2008). Scales to assess the quality of randomized controlled trials: A systematic review. Phys. Ther..

[22] PEDro Physiotherapy Evidence Database (Sitio en Internet). http://www.pedro.org.au/spanish/faq/.

[23] Russo M., Dattola V., De Cola M.C., Logiudice A.L., Porcari B., Cannavò A., Sciarrone F., De Luca R., Molonia F., Sessa E. (2018). The role of robotic gait training coupled with virtual reality in boosting the rehabilitative outcomes in patients with multiple sclerosis. Int. J. Rehabil. Res..

[24] Calabrò R.S., Russo M., Naro A., De Luca R., Leo A., Tomasello P., Molonia F., Dattola V., Bramanti A., Bramanti P. (2017). Robotic gait training in multiple sclerosis rehabilitation: Can virtual reality make the difference? Findings from a randomized controlled trial. J. Neurol. Sci..

[25] Sconza C., Negrini F., Di Matteo B., Borboni A., Boccia G., Petrikonis I., Stankevičius E., Casale R. (2021). Robot-Assisted Gait Training in Patients with Multiple Sclerosis: A Randomized Controlled Crossover Trial. Medicina.

[26] Niedermeier M., Ledochowski L., Mayr A., Saltuari L., Kopp M. (2017). Immediate affective responses of gait training in neurological rehabilitation: A randomized crossover trial. J. Rehabil. Med..

[27] Russo M., Dattola V., Logiudice A.L., Ciurleo R., Sessa E., De Luca R., Bramanti P., Bramanti A., Naro A., Calabrò R.S. (2017). The role of Sativex in robotic rehabilitation in individuals with multiple sclerosis: Rationale, study design, and methodology. Medicine.

[28] Pompa A., Morone G., Iosa M., Pace L., Catani S., Casillo P., Clemenzi A., Troisi E., Tonini A., Paolucci S. (2017). Does robot-assisted gait training improve ambulation in highly disabled multiple sclerosis people? A pilot randomized control trial. Mult. Scler..

[29] Ziliotto N., Lamberti N., Manfredini F., Straudi S., Tisato V., Carantoni M., Melloni E., Secchiero P., Basaglia N., Bernardi F. (2021). Baseline and overtime variations of soluble adhesion molecule plasma concentrations are associated with mobility recovery after rehabilitation in multiple sclerosis patients. J. Neuroimmunol..

[30] Androwis G.J., Sandroff B.M., Niewrzol P., Fakhoury F., Wylie G.R., Yue G., DeLuca J. (2021). A pilot randomized controlled trial of robotic exoskeleton-assisted exercise rehabilitation in multiple sclerosis. Mult. Scler. Relat. Disord..

[31] Puyuelo-Quintana G., Cano-de-la-Cuerda R., Plaza-Flores A., Garces-Castellote E., Sanz-Merodio D., Goñi-Arana A., Marín-Ojea J., García-Armada E. (2020). A new lower limb portable exoskeleton for gait assistance in neurological patients: A proof of concept study. J. Neuroeng. Rehabil..

[32] Łyp M., Stanisławska I., Witek B., Olszewska-Żaczek E., Czarny-Działak M., Kaczor R. (2018). Robot-Assisted Body-Weight-Supported Treadmill Training in Gait Impairment in Multiple Sclerosis Patients: A Pilot Study. Adv. Exp. Med. Biol..

[33] Straudi S., Manfredini F., Lamberti N., Zamboni P., Bernardi F., Marchetti G., Pinton P., Bonora M., Secchiero P., Tisato V. (2017). The effectiveness of Robot-Assisted Gait Training versus conventional therapy on mobility in severely disabled progressIve MultiplE sclerosis patients (RAGTIME): Study protocol for a randomized controlled trial. Trials.

[34] Straudi S., Manfredini F., Lamberti N., Martinuzzi C., Maietti E., Basaglia N. (2020). Robot-assisted gait training is not superior to intensive overground walking in multiple sclerosis with severe disability (the RAGTIME study): A randomized controlled trial. Mult. Scler..

[35] McGibbon C.A., Sexton A., Jayaraman A., Deems-Dluhy S., Gryfe P., Novak A., Dutta T., Fabara E., Adans-Dester C., Bonato P. (2018). Evaluation of the Keeogo exoskeleton for assisting ambulatory activities in people with multiple sclerosis: An open-label, randomized, cross-over trial. J. Neuroeng. Rehabil..

[36] Berriozabalgoitia R., Bidaurrazaga-Letona I., Otxoa E., Urquiza M., Irazusta J., Rodriguez-Larrad A. (2021). Overground Robotic Program Preserves Gait in Individuals with Multiple Sclerosis and Moderate to Severe Impairments: A Randomized Controlled Trial. Arch. Phys. Med. Rehabil..

[37] Drużbicki M., Guzik A., Przysada G., Phd L.P., Brzozowska-Magoń A., Cygoń K., Boczula G., Bartosik-Psujek H. (2021). Effects of Robotic Exoskeleton-Aided Gait Training in the Strength, Body Balance, and Walking Speed in Individuals with Multiple Sclerosis: A Single-Group Preliminary Study. Arch. Phys. Med. Rehabil..

[38] Maggio M.G., De Luca R., Manuli A., Buda A., Foti Cuzzola M., Leonardi S., D’Aleo G., Bramanti P., Russo M., Calabrò R.S. (2022). Do patients with multiple sclerosis benefit from semi-immersive virtual reality? A randomized clinical trial on cognitive and motor outcomes. Appl. Neuropsychol. Adult..

[39] Munari D., Fonte C., Varalta V., Battistuzzi E., Cassini S., Montagnoli A.P., Gandolfi M., Modenese A., Filippetti M., Smania N. (2020). Effects of robot-assisted gait training combined with virtual reality on motor and cognitive functions in patients with multiple sclerosis: A pilot, single-blind, randomized controlled trial. Restor. Neurol. Neurosci..

[40] Israel F., Campbell D., Kahn H., Hornby G. (2006). Metabolic costs and muscle activity patterns during robotic-and therapist-assisted treadmill walking in individuals with incomplete spinal cord injury. Phys. Ther..

[41] Freivogel S., Mehrholz J., Schmalohr D. (2009). Improved walking ability and reduced therapeutic stress with an electromechanical gait device. Rehab. Med..

[42] Iosa M., Morone G., Cherubini A., Paolucci S. (2016). The Three Laws of Neurorobotics: A Review on What Neurorehabilitation Robots Should Do for Patients and Clinicians. J. Med. Biol. Eng..

[43] Maranesi E., Riccardi G.R., Di Donna V., Di Rosa M., Fabbietti P., Luzi R., Pranno L., Lattanzio F., Bevilacqua R. (2020). Effectiveness of Intervention Based on End-effector Gait Trainer in Older Patients with Stroke: A Systematic Review. J. Am. Med. Dir. Assoc..

[44] Shin J.C., Jeon H.R., Kim D., Cho S.I., Min W.K., Lee J.S., Oh D.S., Yoo J. (2021). Effects on the Motor Function, Proprioception, Balance, and Gait Ability of the End-Effector Robot-Assisted Gait Training for Spinal Cord Injury Patients. Brain Sci..

[45] Hidler J., Nichols D., Pelliccio M., Brady K. (2005). Advances in the understanding and treatment of stroke impairment using robotic devices. Top. Stroke Rehabil..

[46] Li Z., Zhang X., Wang K., Wen J. (2018). Effects of Early Mobilization after Acute Stroke: AMeta-Analysis of Randomized Control Trials. J. Stroke Cerebrovasc. Dis..

[47] AVERT Trial Collaboration Group (2015). Efficacy and safety of very early mobilisation within 24 h of stroke onset (AVERT): A randomised controlled trial. Lancet.

[48] Platz T., Kim I.H., Pintschovius H., Winter T., Kieselbach A., Villringer K. (2000). Multimodal EEG analysis in man suggests impairment-specific changes in movement-related electric brain activity after stroke. Brain.

[49] Latash M.L. (2020). On Primitives in Motor Control. Mot. Control.

[50] Morone G., Cocchi I., Paolucci S., Iosa M. (2020). Robot-assisted therapy for arm recovery for stroke patients: State of the art and clinical implication. Expert Rev. Med. Devices.

[51] Selves C., Stoquart G., Lejeune T. (2020). Gait rehabilitation after stroke: Review of the evidence of predictors, clinical outcomes and timing for interventions. Acta Neurol. Belg..

[52] Formaggio E., Storti S.F., Boscolo Galazzo I., Gandolfi M., Geroin C., Smania N. (2013). Modulation of event-related desynchronization in robot-assisted hand performance: Brain oscillatory changes in active, passive and imagined movements. J. Neuroeng. Rehabil..

[53] Basteris A., Nijenhuis S.M., Stienen A.H., Buurke J.H., Prange G.B., Amirabdollahian F. (2014). Training modalities in robot-mediated upper limb rehabilitation in stroke: A framework for classification based on a systematic review. J. Neuroeng. Rehabil..

[54] Porras-Betancourt M., Lilia N.O. (2007). Esclerosis múltiple. Rev. Mex. Neuroci..

[55] Eriksson S., Gard G. (2011). Physical exercise and depression. Phys. Ther. Rev..

[56] Herring M.P., Puetz T.W., O’Connor P.J., Dishman R.K. (2012). Effect of exercise training on depressive symptoms among patients with a chronic illness: A systematic review and meta-analysis of randomized controlled trials. Arch. Intern. Med..

[57] Stroud N.M., Minahan C.L. (2009). The impact of regular physical activity on fatigue, depression and quality of life in persons with multiple sclerosis. Health Qual. Life Outcomes.

[58] Dettmers C., Sulzmann M., Ruchay-Plössl A., Gütler R., Vieten M. (2009). Endurance exercise improves walking distance in MS patients with fatigue. Acta Neurol. Scand..

